# Gradient pooling distillation network for lightweight single image super-resolution reconstruction

**DOI:** 10.7717/peerj-cs.2679

**Published:** 2025-02-07

**Authors:** Zhiyong Hong, GuanJie Liang, Liping Xiong

**Affiliations:** School of Electronics and Information Engineering, Wuyi University, Jiangmen City, Guangdong, China

**Keywords:** Image super-resolution, Computational photography, Image processing

## Abstract

The single image super-resolution (SISR) is a classical problem in the field of computer vision, aiming to enhance high-resolution details from low-resolution images. In recent years, significant progress about SISR has been achieved through the utilization of deep learning technology. However, these deep methods often exhibit large-scale networks architectures, which are computationally intensive and hardware-demanding, and this limits their practical application in some scenarios (*e.g*., autonomous driving, streaming media) requiring stable and efficient image transmission with high-definition picture quality. In such application settings, computing resources are often restricted. Thus, there is a pressing demand to devise efficient super-resolution algorithms. To address this issue, we propose a gradient pooling distillation network (GPDN), which can enable the efficient construction of a single image super-resolution system. In the GPDN we leverage multi-level stacked feature distillation hybrid units to capture multi-scale feature representations, which are subsequently synthesized for dynamic feature space optimization. The central to the GPDN is the Gradient Pooling Distillation module, which operates through hierarchical pooling to decompose and refine critical features across various dimensions. Furthermore, we introduce the Feature Channel Attention module to accurately filter and strengthen pixel features crucial for recovering high-resolution images. Extensive experimental results demonstrate that our proposed method achieves competitive performance while maintaining relatively low resource occupancy of the model. This model strikes for a balance between excellent performance and resource utilization—particularly when trading off high recovery quality with small memory occupancy.

## Introduction

Single-image super-resolution (SISR) represents a long-standing challenge within the field of computer vision. The aim of SISR is to enhance the resolution of a single low-resolution image through a process that yields a restored image of higher resolution. The methodology is designed to recover or augment details, which may have been compromised in the original image as a result of sampling, compression, or other contributing factors. The ultimate goal of SISR is to produce an image that exhibits greater clarity, enhanced fineness, and an increased amount of visual information. In recent years, the advent of deep learning has catalyzed remarkable advancements in super-resolution reconstruction techniques, leading to a steady refinement of performance metrics (*e.g*., Dong et al., 2015; Shi et al., 2016; Kim, Lee & Lee, 2016a; Zhang et al., 2018a, 2021; Chen et al., 2023). Nonetheless, the escalating complexity and depth of these networks have necessitated more powerful hardware capabilities in terms of storage and computational power, which, in turn, constrains their widespread adoption in practical scenarios.

In the contemporary landscape, marked by the ascendancy of autonomous driving and streaming platforms, there exists a burgeoning demand for reliable and efficient image transmission coupled with the delivery of high-fidelity imagery. Given the typically constrained computational resources of these platforms, the imperative to engineer a super-resolution algorithm that is both computationally efficient and effective becomes self-evident.

Dong et al. (2015) have pioneered the field of neural network-based image super-resolution reconstruction with the introduction of the super-resolution convolutional neural network (SRCNN). This approach leverages the convolutional neural networks to effectively enhance image resolution. Consequently, deep learning-based super-resolution algorithms have diversified. Moreover, advances in hardware computing power have led to the development of increasingly larger and deeper neural networks for super-resolution reconstruction (*e.g*., Kim, Lee & Lee, 2016a; Haris, Shakhnarovich & Ukita, 2018; Dosovitskiy et al., 2020; Liang et al., 2021; Chen et al., 2023). For instance, drawing inspiration from VGG-Net (*e.g*., Simonyan, 2015), Kim, Lee & Lee (2016a) have proposed a 20-layer deep network, which outperforms SRCNN in terms of reconstruction quality (*e.g*., Gunasekaran, 2023). Haris, Shakhnarovich & Ukita (2018) have proposed a deep learning-based super-resolution method which boasts over 15.3 million learnable parameters. Despite their high-quality super-resolution performance, these deep and large networks often face practical limitations in terms of available computing resources. Moreover, networks such as Self-Modulation Feature Aggregation Network (SMFANet) (*e.g*., Zheng et al., 2024), Hierarchical Transformer for Efficient Image Super-Resolution (HiT-SR) (*e.g*., Zhang, Zhang & Yu, 2024), Hybrid Attention Transformer (HAT) (*e.g*., Chen et al., 2023) and Image Restoration Using Swin Transformer (SwinIR) (*e.g*., Liang et al., 2021) outperform most of super-resolution (SR) models that rely only on convolutions. These networks all adopt the multi-head self-attention mechanism (*e.g*., Dosovitskiy et al., 2020), and a large number of experimental evidence have confirmed that the multi-head self-attention mechanism shows significant influence in both high-level and low-level visual tasks. However, the deployment of global self-attention mechanisms on current ordinary hardware remains challenging due to the computationally intensive matrix multiplication operations required.

To mitigate computational demands, alleviate equipment strain, and facilitate easier deployment, numerous studies have introduced their respective super-resolution methodologies (*e.g*., Kim, Lee & Lee, 2016b; Tai, Yang & Liu, 2017; Lai et al., 2017; Hui et al., 2019; Liu, Tang & Wu, 2020). For instance, IMDN (*e.g*., Hui et al., 2019) adopts a multi-channel distillation approach to more effectively extracting high-level features through channel separation. Subsequently, RFDN (*e.g*., Liu, Tang & Wu, 2020) revisits IMDN’s design principles and introduces the feature distillation cell (FDC), attaining commendable outcomes; however, its parameter-to-performance ratio remains higher compared to most lightweight SR networks. Meanwhile, some networks, such as (*e.g*., Kim, Lee & Lee, 2016b; Lai et al., 2017; Tai, Yang & Liu, 2017), leverage recursive learning strategies to curtail the number of parameters. Nonetheless, these techniques come at the cost of significantly elongated inference times, thus compromising the overall inference efficiency of the models. On another front, works like Edge-oriented Convolution Block for Real-time Super Resolution (ECBSR) (*e.g*., Zhang, Zeng & Zhang, 2021) and Real-Time 4K Image Super-Resolution (RT4KSR) (*e.g*., Zamfir, Conde & Timofte, 2023) employ reparameterization techniques, which not only decrease the parameter count during inference but also markedly expedite the model’s operational speed. Yet, despite these gains, these models exhibit marginally inferior SR performance, thereby suggesting a potential trade-off between performance and speed within the model architecture. A quest for a better-balanced approach that optimizes both aspects remains an open research question in the domain of single-image super-resolution.

To identify an optimal balance between complexity and performance, we introduce a streamlined and potent model named gradient pooling distillation network (GPDN). Not only does GPDN demonstrate outstanding super-resolution prowess, but it also boasts an equally remarkable lightweight parameter. The core component of the proposed model is the Feature Mixture Distillation module (FMDM), which consists of two sub-modules: the Pooling Distillation module (PDM) and the Channel Attention module (CAM) (*e.g*., Chen et al., 2023). Our GPD module amalgamates the salient features of IMD (*e.g*., Hui et al., 2019) and FASM (*e.g*., Sun et al., 2023). The fundamental operational sequence of the GPD module involves the segregation of feature maps extracted from identical locations, followed by distinct pooling and convolution operations, culminating in the amalgamation into a novel feature map. This process yields feature maps across multiple dimensions without an increase in the parameter count.

The PDM is predicated on this partitioned pooling mechanism, with the distinction that it employs IMD’s separation methodology, whereby the feature map is partitioned after each pooling layer. Additionally, our pooling strategy deviates from SAFM’s single-step approach by incrementally pooling downsamples. This incremental pooling not only further condenses the parameter count but also enhances operational efficiency. When juxtaposed with certain ultra-compact models, our approach demonstrates a minimal compromise in reconstruction precision.

The principal contributions of this study are outlined as follows:

1. We introduce an ultra-lightweight GPD module (GPD), which integrates the strengths of IMD and SAFM, enabling the efficient parsing of both upper and lower feature maps with an exceptionally compact parameter footprint. This novel module demonstrates particular efficacy in the context of lightweight SR networks.

2. Building upon GPD, we devise a novel CAM, which significantly enhances the representational capacity of the network.

3. We subject the proposed GPDN framework to rigorous quantitative and qualitative assessments on publicly accessible datasets. The results attest to the remarkable SR performance attainable by our GPDN, even with a smaller number of parameters, thus substantiating its effectiveness and efficiency in the realm of single-image super-resolution.

## Related work

**Super-resolution reconstruction algorithm based on deep learning.** In recent years, on the one hand, the continuous advancement of science and technology has rendered images an increasingly pivotal medium for human information conveyance. To satisfy the demand for high-quality images, deep learning-based super-resolution (SR) reconstruction algorithms have emerged as the predominant solution. Dong et al. (2015) introduced SRCNN (*e.g*., Dong et al., 2015), which is the first application of convolutional operations in neural networks for SISR. VDSR (*e.g*., Kim, Lee & Lee, 2016a) was the first to employ a global residual deep network for SR. This demonstrates that network depth correlates positively with SR performance. SRResNet (*e.g*., Ledig et al., 2017), which integrates deep residual learning with sub-pixel convolution, significantly enhances SR capabilities. Subsequently, EDSR (*e.g*., Lim et al., 2017) refined its predecessor, SRResNet (*e.g*., Ledig et al., 2017), by eliminating batch normalization (BN) layers deemed ineffectual for super-resolution tasks. This enhancement makes EDSR win the top position in the NTIRE 2017 Super-Resolution Challenge (*e.g*., Timofte et al., 2017). However, the parameter count of its baseline model was stupendous, amounting to about 43 million. RDN (*e.g*., Zhang et al., 2018b) leverages local dense connections, efficiently utilizing all convolutional layers and fusing features through both local and global feature fusion. RCAN (*e.g*., Zhang et al., 2018a) introduced the channel attention mechanism, focusing network resources on inter-channel communication and enhancing learning capabilities. On the other hand, highlighted by the successful application of Vision Transformer (ViT) (*e.g*., Dosovitskiy et al., 2020), Transformer spurred the development of numerous SR networks incorporating ViT technology. For instance, SwinIR (*e.g*., Liang et al., 2021), a network composed of multiple Swin Transformer layers with residual connections, achieves accurate SR with fewer parameters. HAT (*e.g*., Chen et al., 2023) combines the self-attention and channel attention mechanisms of Swin Transformer, harnessing the benefits of global self-attention and traditional convolutional local fitting, and is recognized as the current state-of-the-art SR model. Despite their impressive SR results, the computational demands of these models present challenges for deployment on real-world edge devices, such as smart phones, tablets, *etc*.

**Lightweight efficient image super-resolution.** In the pursuit of deploying deep learning-based super-resolution (SR) algorithms on resource-constrained edge devices, many efficient approaches have been proposed. Among them, notably, Fast Super-Resolution Convolutional Neural Networks (FSRCNN) (*e.g*., Dong, Loy & Tang, 2016), an improvement over SRCNN (*e.g*., Dong et al., 2015), not only accelerates processing speed but also enhances SR output quality. Efficient Sub-Pixel Convolutional Neural Network (ESPCN) (*e.g*., Shi et al., 2016) introduced sub-pixel convolution, a rapid and computationally efficient upsampling technique devoid of explicit convolution operations, which yields superior SR results. IDN (*e.g*., Hui, Wang & Gao, 2018) proposed an Information Distillation Block that adaptively partitions features into two distinct components. Further along this line of inquiry, IMDN (*e.g*., Hui et al., 2019) puts forth a lightweight Feature Information Distillation Network. RFDN (*e.g*., Liu, Tang & Wu, 2020), following critical reflection on the IMDN (*e.g*., Hui et al., 2019) architecture, devised an even more lightweight network with enhanced SR performance, ultimately securing victory in the AIM2020-ESR (*e.g*., Zhang et al., 2020) competition. Blueprint Separable Residual Network (BSRN) (*e.g*., Li et al., 2022), adopting a structure reminiscent of (*e.g*., Haase & Amthor, 2020), incorporated the Blueprint Shallow Residual Block, a lightweight and efficacious residual module. Residual Local Feature Network (RLFN)Reconstructed Convolution module based Look-up table (RCLUT) (*e.g*., Kong et al., 2022) harnessed a three-layer convolutional architecture with row-wise residual feature learning to refine feature aggregation, complemented by a novel multi-stage priming training regimen. Pan (*e.g*., Zhao et al., 2020) introduced Pixel Attention (PA), a mechanism akin to spatial and channel attention, wherein the distinguishing characteristic of PA lies in generating not 1D attention feature vectors or 2D attention maps, but rather 3D matrices as attention features. SAFMN (*e.g*., Sun et al., 2023) proposed a Spatially Adaptive Feature Modulation (SAFM) method, implementing a multi-scale feature modulation mechanism capable of dynamically selecting salient features based on non-local feature correlations. Lastly, RC-LUT (*e.g*., Liu et al., 2023) initially employs a proposed SR network to construct a look-up table (LUT), subsequently utilizing this LUT for SR operations. Consequently, the network’s inference phase relies primarily on CPU computations, thus imposing smaller computational power requirements. The aforementioned methods have made significant advancements in enhancing the efficacy of super-resolution algorithms. However, there still exists potential for further optimization, particularly in achieving a better balance between reconstruction performance and model efficiency.

## Method

In this section, we detail our proposed lightweight single-image super-resolution model, called gradient pooling distillation network (GPDN), and dissect its core components.

### Network architecture

**Figure 1:**
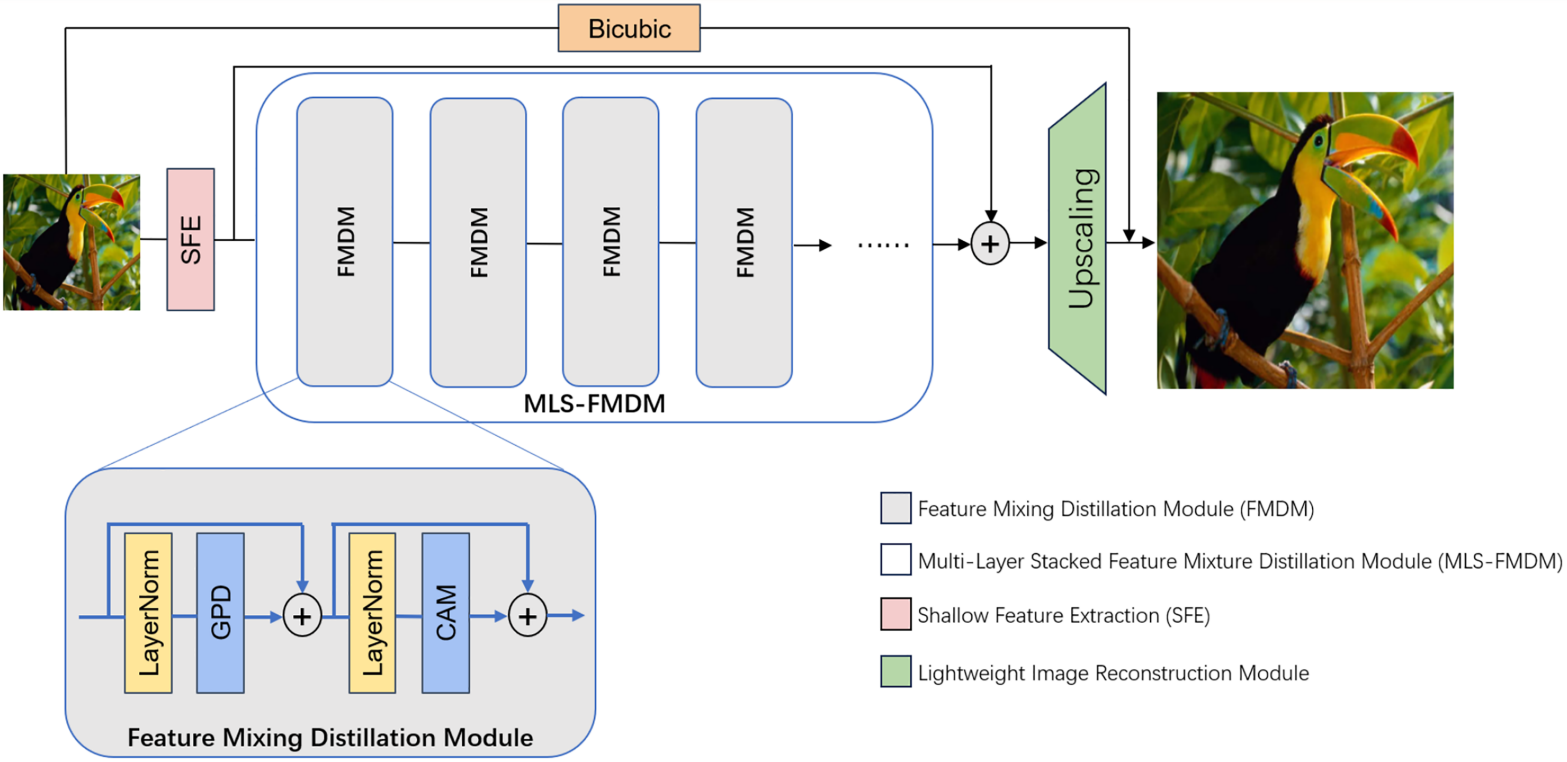
Presents the overall architectural layout of our proposed GPDN. Initially, the low-resolution (LR) input image is projected onto a higher-dimensional feature space *via* a 3 × 3 convolutional layer. Subsequently, the network undergoes a sequential cascade of feature mixture distillation modules (FMDMs) each dedicated to refining and fusing the extracted features. Each FMDM encompasses a Gradient Pooling Distillation module (GPD), a feature channel attention module (CAM), and a pair of skip connections, all of which collaboratively engage in the extraction and integration of the feature representation.

As depicted in Fig. 1, our proposed network architecture comprises three primary components: Shallow Feature Extraction (SFE) module, Nonlinear Mapping module incorporating Multi-Layer Stacked Feature Mixture Distillation module (MLS-FMDM), and lightweight image reconstruction module. Here, the SFE module is used to convert low-resolution images into high-dimensional features; MLS-FMDM refines and distills high-dimensional features; and the lightweight image reconstruction module reconstructs the previously distilled features into a high-resolution image.

In this network, the Shallow Feature Extraction (SFE) module is a 3 × 3 convolutional layer, which aims to convert the input low-resolution image into a higher-dimensional feature representation, denoted as ${F_0}$, which is essential for subsequent network operations. This transformation is facilitated through a local linking operation. The SFE module can be articulated as follows:

(1) $${F_0} = {f_{sfe}}\left( {{I_{LR}}} \right).$$

Here, the function ${f_{sfe}}\left( \cdot \right)$ denotes a 3 × 3 convolutional layer, and ${I_{LR}}$ signifies the input low-resolution image.

Subsequently, we employ an innovative Multi-Layer Stacked Feature Mixture Distillation module (MLS-FMDM) to further refine and optimize the initial high-dimensional features ( ${F_0}$), thereby generating deep features suitable for network upsampling operations aimed at high-resolution image reconstruction. The FMDM is architecturally constituted by a Gradient Pooling Distillation module (GPD) and a Channel Attention module (CAM). Here, both GPD and CAM will be introduced later.

Notably, our design incorporates global residual connections throughout the entire network architecture, which can ensure seamless information flow between all layers and accelerate the training process. Moreover, we embed dense residual connections within the internal configuration of the MLS-FMDM, allowing the network to preserve local detail alongside feature extraction. This promotes feature sharing and propagation, enhancing both the performance and efficiency of the network. We denote the proposed MLS-FMDM as follows:

(2) $${F_n} = f_{{\rm FMDM}}^n\left( {f_{{\rm FMDM}}^{n - 1}\left( { \ldots f_{{\rm FMDM}}^0\left( {{F_0}} \right) \ldots } \right)} \right),$$

(3) $$f_{FMDM}^0\left( {{F_0}} \right) = {f_{CAB}}\left( {LN({f_{GPD}}\left( {{F_0}} \right) + {F_0})} \right) + {F_0}.$$

In these two equations, ${f_{FMDM}}\left( \cdot \right)$ symbolizes the FMDM layer, ${f_{GPD}}\left( \cdot \right)$ denotes the GPD layer, $LN\left( \cdot \right)$ represents layer normalization, ${f_{CAB}}\left( \cdot \right)$ signifies the CAM layer, and ${F_n}$ denotes the feature map corresponding to the output of the $n$th FMDM, respectively.

Finally, to ensure model efficiency, we employ a streamlined upsampling technique that amalgamates the shallow feature map ${F_0}$ with the deep feature map ${F_n}$, subsequently resizing to the desired dimensions of the high-definition (HD) image. This component comprises a 3 × 3 convolutional layer followed by a pixel shuffling layer. We refer to the resultant output ${I_{SR}}$ as the super-resolution image.

(4) $${I_{SR}} = {f_{up}}\left( {{F_0} + {F_n}} \right).$$

Here, ${I_{SR}}$ represents the final, reconstructed high-definition (HD) image, while ${f_{up}}$ denotes the upsampling module employed in the process.

In these whole processes, the low-resolution image stays the same size in the first two modules, and only through the third module the resultant image would be enlarged by a factor of 2, 3, or 4.

### Gradient pooling distillation module

In order to enhance the model’s dynamic modeling ability and hierarchical distillation efficiency, especially in the face of complex datasets, the multi-head pooling strategy proposed in SAFM (*e.g*., Sun et al., 2023) is considered in our approach. This strategy can capture multi-scale information more efficiently and enhance the sensitivity of the model to fine details. At the same time, we also pay attention to the design principle of the hierarchical distillation architecture from RFDB (*e.g*., Liu, Tang & Wu, 2020), which enhances the feature representation of the model by decomposing and reorganizing the features. Based on the above two lightweight architectural choices, we designed the GPD module.

**Figure 2:**
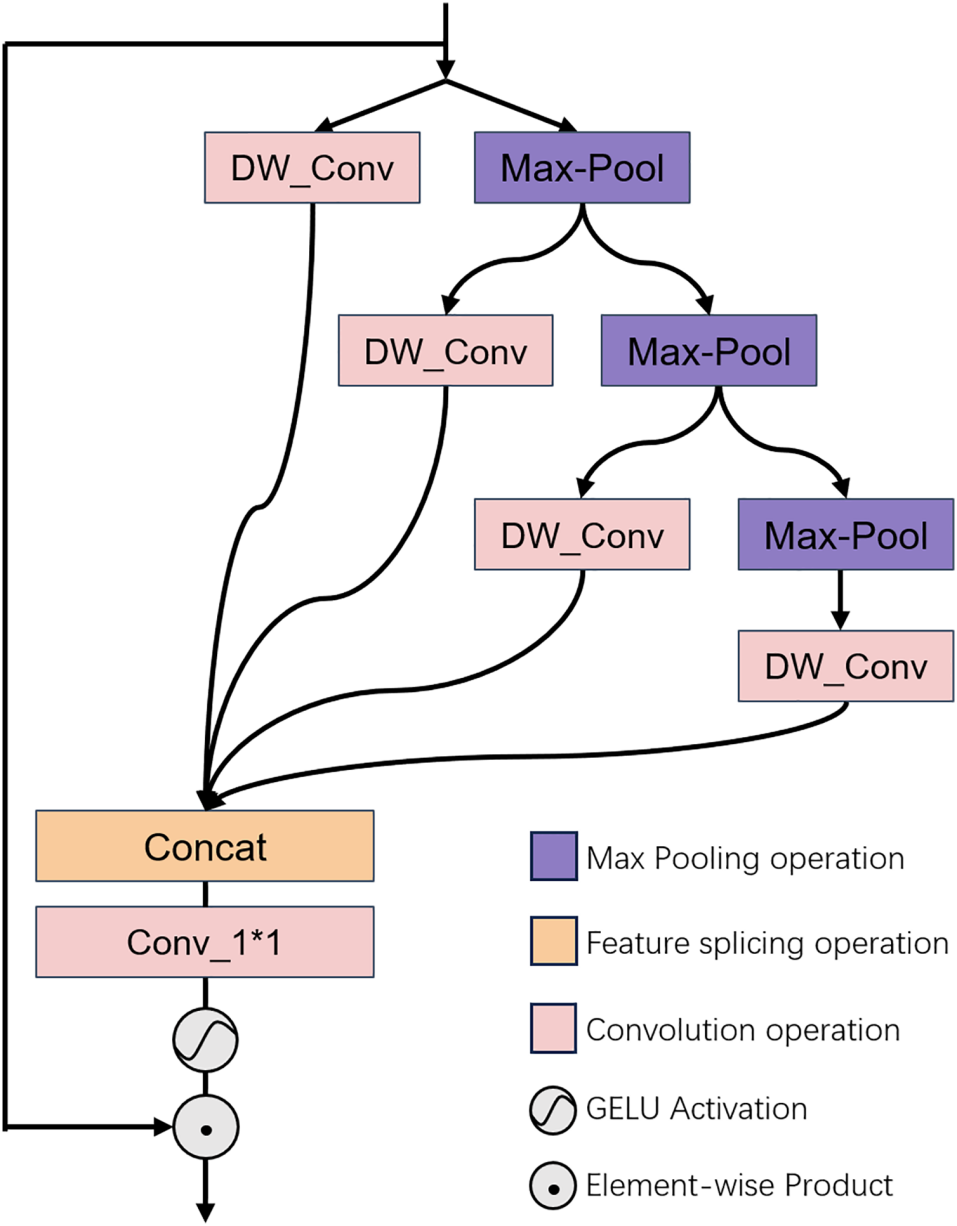
Illustrates the architectural configuration of the proposed GPD module. Illustrates the architectural configuration of the proposed GPD module, which is designed to conduct multi-dimensional distillation of the input features. For a detailed explanation, please refer to “Channel Attention Module”.

The GPD architecture is depicted in Fig. 2. This module is predicated on the concept of multi-head hierarchical pooling. It executes feature extraction through a hierarchical pooling approach, and processes the input data at various levels. At each stratum, features are handled independently, to ensure that the model attends to information across different scales. This hierarchical approach not only bolsters computational efficiency but also facilitates the model’s comprehension and learning of the image’s textural structure. The features, once processed, are consolidated through a 1 × 1 convolution to produce a distilled feature space map that encapsulates multi-scale information.

Concretely, we commence by performing a bisection operation on the normalized input feature channels, dividing them into two constituent parts. One portion is preserved intact, while the remaining portion undergoes further feature distillation procedures. The retained portion is subjected to a layer of deep convolution for direct feature manipulation. Conversely, the other portion is independently subjected to a pooling operation, followed by another bisection of the feature channels and repetition of the preceding steps. This iterative process can be outlined as follows:

(5) $$\eqalign{&F_{d1},F_{c1}=Split_1\left({\rm LN}\left(F_i\right)\right),\\& F_{d2},F_{c2}=Split_2\left(P_{{1\over k}\downarrow}\left(F_{c1}\right)\right),\\& F_{d3},F_{c3}=Split_3\left(P_{{1\over k}\downarrow}\left(F_{c2}\right)\right),\\& F_{d4}=P_{{1\over k}\downarrow}\left(F_{c3}\right),\\& {\hat F_1}= DW\_{\rm Conv}_{3\times 3}\left(F_{d1}\right),\\& {\hat F_i}=I_{\uparrow}\left(DW\_{\rm Conv}_{3\times 3}\left(F_{di}\right)\right),2\leq i \leq 4.}$$

In this context, $LN\left( \cdot \right)$ indicates layer normalization, while $Spli{t_j}$ denotes the operation for separating the feature channels at the $jth$ layer. ${F_i}$ is denoted as the ith layer feature. ${F_{dj}}$ and ${F_{cj}}$ correspond to the distillation and coarse features extracted at the $jth$ layer, respectively. The notation ${P_{{1 \over k} \downarrow }}$ indicates a pooling down-sampling operation with a factor of $k{\rm \; }\left( {k = 2,{\rm \; \; }4,{\rm \; \; }8} \right)$. Different $k$ corresponds to different GPDN versions. $D{W_ - }{\rm Con}{{\rm v}_{3 \times 3}}\left( \cdot \right)$ refers to a 3 × 3 depthwise convolution, and ${I_ \uparrow }\left( \cdot \right)$ represents the operation of upsampling the feature size back to that of the original image using linear interpolation.

Subsequently, the feature values yielded by each distillation operation are concatenated, yielding ${\hat F_i}{\rm \; }\left( {1 \le i \le 4} \right)$ following a layer of 1 × 1 convolution. Thereafter, we employ the GELU activation function to inject nonlinearity into the model, thereby facilitating the estimation of the attention distribution. The comprehensive process can be encapsulated as follows:

(6) $$\matrix{ {\hat F = {\rm Con}{{\rm v}_{1 \times 1}}\left( {{\rm Concat}\left( {\left[ {{{\hat F}_1},{{\hat F}_2},{{\hat F}_3},{{\hat F}_4}} \right]} \right)} \right)} \cr {\bar F = \phi \left( {\hat F} \right) \odot {F_0}.}\hfill \cr }$$

In this framework, ${\rm Concat}\left( \cdot \right)$ denotes the concatenation of features, ${\rm Con}{{\rm v}_{1 \times 1}}\left( \cdot \right)$ represents the application of a 1 × 1 convolution, $\phi \left( \cdot \right)$ refers to the employment of an activation function, and $\odot$ signifies element-wise multiplication.

### Channel attention module

After being distilled and processed by the GPD module, these features shall enter the CAM module for extraction once again.

**Figure 3:**
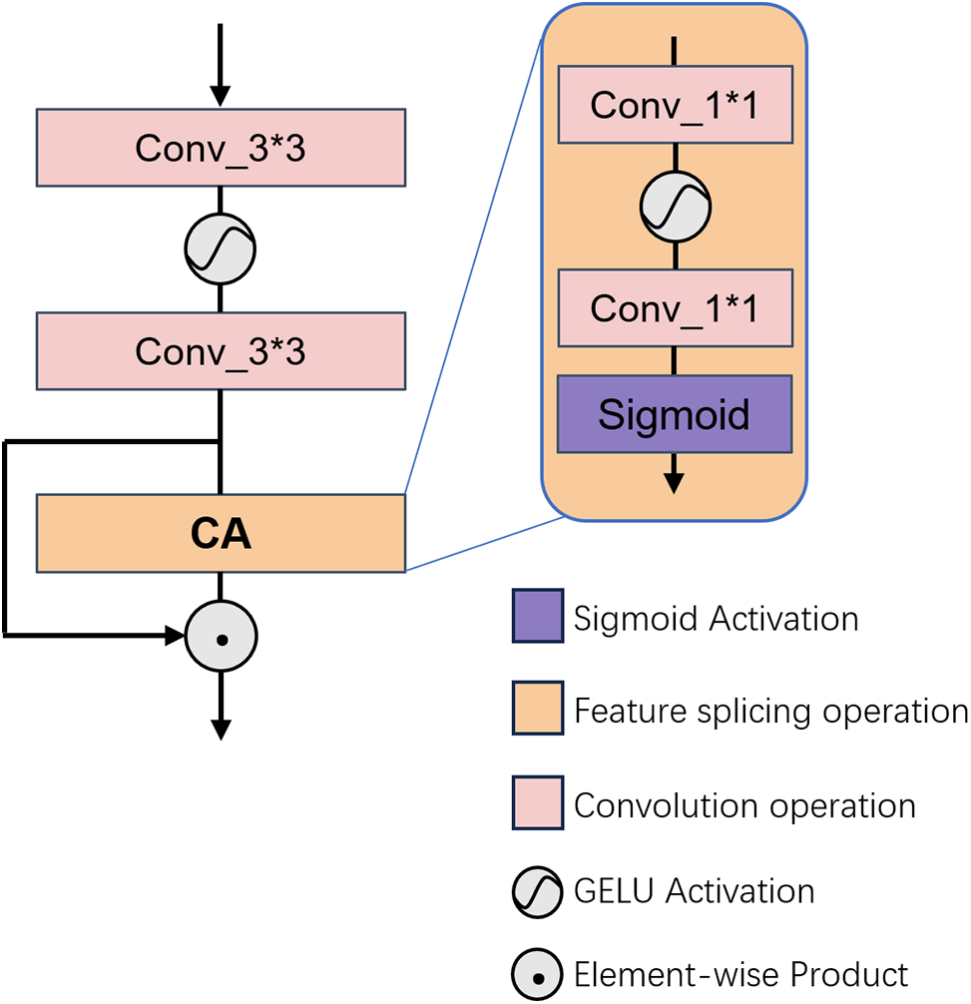
Illustrates the architectural configuration of the proposed CAM.

The CAM architecture is depicted in Fig. 3. As previously discussed, the fundamental role of the CAM in FMDM is to accentuate the significance of salient pixels across dimensions through meticulous filtering of key features. To augment the contextual integration capabilities of our model, we incorporate the CAM proposed by HAT (*e.g*., Chen et al., 2023). This CAM consists of a pair of 3 × 3 convolutions, two 1 × 1 convolutions, and a pooling layer. In the CAM’s initial stage, channel compression and expansion are achieved *via* the consecutive application of two 3 × 3 convolutional layers, followed by an initial screening of the feature channels. Subsequently, a pooling layer is utilized to condense the spatial dimensionality of the feature map, after which two layers of 1 × 1 convolutions are employed to compute channel weights. Ultimately, the sigmoid function is applied to finalize the refinement of the feature map.

## Experiments

In this section, we describe the datasets utilized for training and evaluation, then we elucidate the methodology’s implementation process. By employing the state-of-the-art methods, we conduct both quantitative and qualitative analyses to show the efficacy of our proposed algorithm.

### Dataset and implementation details

**Datasets and metrics.** According to previous research (*e.g*., Zamfir, Conde & Timofte, 2023; Sun et al., 2023; Zhao et al., 2020), we selected 800 HD images in the DIV2K (*e.g*., Agustsson & Timofte, 2017) dataset and 2,650 HD images in the Flickr2K (*e.g*., Lim et al., 2017) dataset and merged them as the training dataset. In this process, we perform bicubic interpolation downsampling on these HD images to generate corresponding low-resolution (LR) images. In our tests, we selected four widely recognized benchmark datasets, namely Set5 (*e.g*., Bevilacqua et al., 2012), Set14 (*e.g*., Zeyde, Elad & Protter, 2012), BSD100 (*e.g*., Timofte, De Smet & Van Gool, 2015), and Urban100 (*e.g*., Huang, Singh & Ahuja, 2015). The general information of all used datasets is shown in Table 1.

**Table 1:** Short description of all used dataset.

Dataset	Amount	Format	Short description
Set5 (Bevilacqua et al., 2012)	5	PNG	Images used for testing, including babies, birds, butterflies, heads, and women
Set14 (Zeyde, Elad & Protter, 2012)	14	PNG	Images used for testing, including people, animals, insects, *etc*.
BSD100 (Timofte, De Smet & Van Gool, 2015)	100	PNG	Images used for testing, including animals, buildings, food, *etc*.
Urban 100 (Huang, Singh & Ahuja, 2015)	100	PNG	Images used for testing, including cities, cities, structures, *etc*.
DIV2K (Agustsson & Timofte, 2017)	1,000	PNG	Each image has 2K pixels and includes the environment, vegetation, animals, *etc*.
Flickr2K (Lim et al., 2017)	2,650	PNG	High definition images of people, animals, landscapes, *etc*.

To measure the effect of image restoration, we used peak signal-to-noise ratio (PSNR) and structural similarity index (SSIM) as evaluation indexes. Also, learned perceptual image patch similarity (LPIPS) (*e.g*., Zhang et al., 2018c) was used as a supplementary evaluation index.

PSNR is a quantitative index used to objectively evaluate image quality, which is used to measure the degree of similarity or difference between the original image and the reconstructed image. The basic calculation formula is as follows:

(7) $$MSE={{1}\over{mn}}\sum\nolimits_{i=1}^m\sum\nolimits_{j=1}^n\left[I_{ij}-K_{ij}\right]^2$$

(8) $$PSNR = 10 \cdot {\log _{10}}\left( {\displaystyle{{MAX_I^2} \over {MSE}}} \right).$$

Here $I$ means the original image, $K$ is the reconstructed image, $m$ and $n$ represent image height and width, respectively, ${I_{ij}}$ and ${K_{ij}}$ are the $\left( {i,j} \right)$ pixel values of images $I$ and $K$ respectively, $MA{X_I}$ refers to the possible maximum pixels in the image.

Similar to PSNR, SSIM also compares the difference between the processed image and the original image, however, it focuses more on simulating the perception of the image structure by the human eye. It is calculated as follows:

(9) $$SSIM\left( {x,y} \right) = \displaystyle{{\left( {2{\mu _x}{\mu _y} + {C_1}} \right)\left( {2{\sigma _{xy}} + {C_2}} \right)} \over {\left( {\mu _x^2 + \mu _y^2 + {C_1}} \right)\left( {\sigma _x^2 + \sigma _y^2 + {C_2}} \right)}}.$$

Among them, the ${\mu _x},{\mu _y}$ are the average of $x$ and $y$ pixel, $\sigma _x^2,\sigma _y^2$ are their variance, ${\sigma _{xy}}$ are their covariance. The constants ${C_1}$ and ${C_2}$ are positive constants used to avoid instability caused by denominators approaching zero, and they are usually associated with the maximum pixel value of the image and a small constant.

To perform these evaluations, we first convert the image to YcbCr color space and then calculate the PSNR and SSIM in the Y channel.

Regarding the supplementary evaluation index LPIPS, it is a learning-based perceptual image patch similarity indicator, which is mainly used to evaluate the perceptual quality of images. This indicator uses convolutional neural networks (CNN) to perform feature extraction operations on local patches of images and calculate the similarity score between patches.

**Detailed rules for implementation.** Following the previous work (*e.g*., Bilecen & Ayazoglu, 2023), which is the winner of NTIRE RTSR Challange Track 2 (x3 SR), the number of GPD modules and the number of feature channels are set as 8 and 32, respectively. Moreover, we use the KneeLR scheduler in Bilecen & Ayazoglu (2023) to dynamically the learning rate. We set the base learning rate to $5 \times {10^{ - 4}}$ and the minimum learning rate is $5 \times {10^{ - 6}}$. The number of model training iterations is 1,000. To further refine the content of the experimental dataset, we adopt the slicing operation. To increase the diversity of the training data, we also used random horizontal flips and 90 rotations. All our experimental operations are performed in the hardware environment equipped with NVIDIA GeForce RTX 3090 GPU, and PyTorch deep learning framework is adopted to complete the experiment programming and model training work.

### Model analysis

**Distillation pooling rate experiment.** The purpose of this experiment is to explore the most suitable distillation pooling rate for the GPD module in the most suitable GPDN network. We set four different pooling rates for comparison, which are two times, four times, eight times of pooling downsampling per layer, and an extreme case, that is, only two pixels downsampling per layer. We use GPDN_2, GPDN_4, GPDN_8, and GPDN_2p to represent the downsampled versions of the network with different multipliers, with the underlining after the underscore indicating the downsampling multipliers.

From the results in Table 2, we know that GPDN_8 and GPDN_2p perform better in Set5 and Set14 datasets. Especially, for GPDN_2p, PSNR index and SSIM index are 0.2527dB and 0.0044 higher than those for GPDN_2 in Set5. However, GPDN_2 has the best performance on the two benchmark datasets of B100 and Urban100, both of which show good performance in PSNR and SSIM indicators. Therefore, we speculate that the increase of the sampling rate will increase the degree of GPDN network to learn the characteristics of the data set, but will affect the generalization ability of GPDN.

**Table 2:** Effect of distillation pooling rate on the network.

Variant	#Params (K)	Set5	Set14	B100	Urban100
AdaptiveMaxPool level → 2	103	31.6907/0.8836	28.3182/**0.7747**	**26.9763**/**0.7048**	**24.9390**/**0.7441**
AdaptiveMaxPool level → 4	103	31.8409/0.8865	28.3167/0.7740	26.8969/0.7025	24.7428/0.7367
AdaptiveMaxPool level → 8	103	31.9082/0.8875	**28.3471**/0.7745	26.9736/0.7042	24.8374/0.7399
AdaptiveMaxPool level → 2 pixel	103	**31.9434**/**0.8880**	28.2967/0.7728	26.9123/0.7030	24.8050/0.7383

**Note:**

This experiment is a study on the benchmark dataset with upsampling factor ×4, and the evaluation metrics are PSNR↑ and SSIM↑, respectively. The best metrics are identified in bold with scores.

Ablation experiments of the main module. To gain a deeper understanding and accurately evaluate the performance of our proposed GPDN model, we conduct an ablation study on the main components of the model. This study is to adopt the GPDN_8 version of the network architecture. This version is a GPDN, which adopts max pooling with 8 * 8 filters and stride 2. And the training is carried out on the untrimmed training dataset. We use the standard dataset with an upsampling factor of ×4 for testing, covering the two datasets Set14 and B100.

To verify the effectiveness of the GPD module, we first remove the GPD module. Based on the results in Table 3 we can observe that the performance of the model is significantly reduced in the absence of the GPD module. Specifically, on the Set14 and B100 datasets, the PSNR value is reduced by 0.21272 and 0.0524 dB, respectively, which highlights the importance of the GPD module in improving the performance. After removing the max pooling distillation step in the GPD module, we notice a noticeable degradation in the performance of super-resolution compared to the baseline model. This shows that the quality of super-resolution can be effectively improved by introducing a pooling distillation step, even without adding any trainable parameters. This is due to the fact that the design of this structure is aimed at reducing the data dimension layer by layer and highlighting the main feature information within the data. Each layer of the network is capable of extracting the most representative portion of the data. As a result, the model can concentrate on these crucial features and disregard some subtle changes and noise. Furthermore, the required Parameters and FLOPs are not increased. This point verifies the effectiveness of the layer-by-layer pooling distillation strategy we adopted.

**Table 3:** Ablation experiments of the main module.

Variant	#Params (K)	#FLOPs (G)	Set14	B100
Base	102.7	6.432	**28.2262/0.7721**	**26.8851/0.7021**
GPD → None	91.208	5.811	28.0990/0.7697	26.8327/0.6995
CAB → None	26.288	1.586	27.8113/0.7587	26.7132/0.6939
GPD → None maxpool	102.7	6.432	28.2149/0.7717	26.8839/0.7011
maxpool → Conv	397.7	11.587	28.4749/0.7733	26.8799/0.7026
GPD.dim → 64	372.3	23.606	28.4432/0.7726	26.8706/0.7018
GPD.dim → 72	466	19.577	28.4453/0.7728	26.8718/0.7019
GPD.dim → 128	1,412	90.162	28.3433/0.7721	26.8712/0.7014

**Note:**

This experiment is a study on the benchmark dataset with upsampling factor ×4, and the evaluation metrics are PSNR↑ and SSIM↑, respectively. The best metrics are identified in bold with scores. Params represents the number of parameters the network can learn↓ and FLOPs represents the network.

After replacing the max-pooling distillation in the GDP module with convolutional distillation, we can clearly observe a significant increase in both model parameters and computational load. Although the enhancement in super-resolution quality is evident, the parameter count has escalated by almost four times. We argue that trading nearly quadruple storage space for marginal improvements in super-resolution performance does not offer compelling value. Furthermore, we have increased the number of feature channels in the model, gradually increasing from the original 32 to 128. Experimental results indicate that the super-resolution reconstruction quality of the model does not continuously improve with the increase in the number of feature channels. When the number of channels increased to 72, there was indeed an improvement in super-resolution quality, but the number of parameters also increased significantly. Furthermore, when the number of feature channels was increased to 128, the model exhibited overfitting during the training process, leading to a decrease in super-resolution quality and reduced generalization. Based on these findings, we believe that having 32 feature channels can strike a good balance among the number of parameters, computational load, and super-resolution quality.

**Figure 4:**
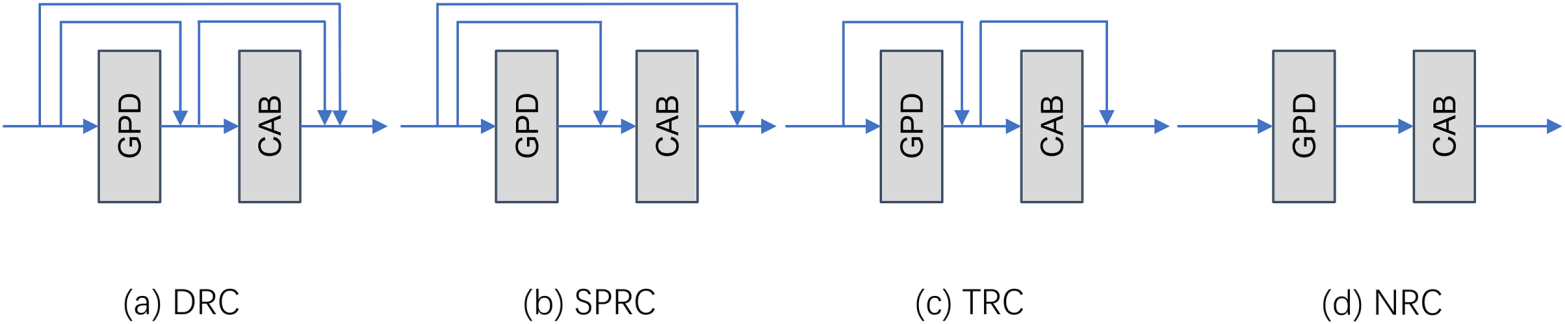
Demonstration of the four local residual link mode. (A) Dense residual connection, (B) single point residual connection, (C) traditional residual connection, (D) none residual connection.

**Effect of local residual link mode on the network.** After the main frame of the network is determined, we further explore the impact of different types of local residual connections on the final super-resolution accuracy. We experimented a total of four kinds of local residual as shown in Fig. 4. In the experiments, we selected four different local residual structures for comparison. They are dense residual connection (DRC), single point residual connection (SPRC), traditional residual connection (TLR), and no residual connection (NRC). These experiments are all performed under the GPDN_8 model version using the original untrimmed dataset.

It can be found from the data in Table 4 that on the Set14 benchmark data set, TLR performs the best. Its PSNR value is 0.3582 dB higher than the worst DRC, 0.1757 dB higher than the second highest SPRC, and 0.0007 higher than the SSIM index. However, in this dataset Validation set for DlV2K, SPRC slightly outperforms TLRC in terms of PSNR and SSIM metrics, although this advantage is not significant. In addition, the performance of NRC on the two datasets Set14 and DIV2K.val does not show any advantage. The results of this experiment also demonstrate the effectiveness of residual connections in the GPDN model.

**Table 4:** Effect of local residual link mode on the network.

Variant	#Params (K)	Set14	DIV2K.val
DRC	103	27.8680/0.7610	29.5341/0.8108
SPRC	103	28.1805/0.7714	**30.1019/0.8272**
TLRC	103	**28.2262/0.7721**	30.0394/0.8267
NRC	103	28.1532/0.7703	30.0083/0.5257

**Note:**

This experiment is a study on the benchmark dataset with upsampling factor ×4, and the evaluation metrics are PSNR↑ and SSIM↑, respectively. The best metrics are identified in bold with scores.

**Training dataset segmentation experiments.** To speed up the model training efficiency, we consider the segmentation of the training dataset. In the training of the proposed GPDN model, the cropped versions of the traditional DIV2K and Flickr2K are used. In this version, after combining the above two data sets, the software is used to cut them into several parts in a certain proportion. From the data in Table 5, we can see that training with the cropped training data set will obtain better training results than training with the uncropped training data set. For example, there is a big gap between the results trained on Flickr2k data and Flickt2K_sub data set. The PSNR index on Set14 is 0.1026 dB lower, and the effect of training on DIV2K is also worse than that on DIV2K_sub. The PSNR index on B100 is 0.2661 lower. The experiments fully prove that the quality of super-resolution can be improved by cutting and expanding the dataset without increasing any learnable parameters in the network.

**Table 5:** Training dataset segmentation experiments.

Variant	#Params (K)	Set14	B100
Flickr2K (Lim et al., 2017)	103	28.2006/0.7716	26.8500/0.7014
Flickr2K_sub	103	28.3032/0.7741	26.9227/0.7035
DIV2K (Agustsson & Timofte, 2017)	103	28.0363/0.7665	26.8191/0.6990
DIV2K_sub	103	28.3024/0.7737	26.9081/0.7029
DF2K	103	28.2262/0.7721	26.8851/0.7021
DF2K_sub	103	28.3471/0.7745	26.9736/0.7042

**Note:**

This experiment is the result of validation on GPDN_8, a dataset with upsampling factor ×4. Flicker2K represents the original dataset, Flickt2K_sub represents the cropped dataset. DIV2K represents the original dataset, DIV2K_sub represents the cropped dataset, DF2K is the collection of Flickr2K+DIV2K, and DF2K represents the cropped dataset.

### Comparison with state-of-the-art methods

It is obvious from Tables 6 and 7 that the number of learnable parameters of our proposed GPRN is significantly less than that of other SR methods we compared. At the same time, the demand for computing power is also close to or even lower than the algorithms we compared. Among them, our model can save up to 97.66% of the computing power on average. However, in terms of the number of learnable parameters, the other algorithms we compared have on average 5 times more parameters than our model.

**Table 6:** Comparison with state-of-the-art methods in the Set5 dataset and the Set14 dataset. In this experiment, all are the results of dataset validation with upsampling factor ×4. Our models are trained with the cropped training dataset (DF2K_sub). GPDN_2 and GPDN_8 are our proposed models.

Methods	#FLOPs (G)	#Params (K)	Set5		Set14	
			PSNR (dB,↑)	SSIM (↑)	PSNR (dB,↑)	SSIM (↑)
Bicubic	–	–	28.76	0.8113	26.72	0.8652
RT4KSR-XXXL (Zamfir, Conde & Timofte, 2023)	3.46 (−57%)	110.4 (+7.5%)	30.45	0.8610	28.04	0.7814
CARN (Ahn, Kang & Sohn, 2018)	426 (+6,627%)	1,592 (+1,450%)	32.13	0.9590	28.60	0.7806
LapSRN (Lai et al., 2017)	370 (+5,752%)	813 (+692%)	31.54	0.8850	28.19	0.7720
SwimIR (Liang et al., 2021)	373 (+5,800%)	878 (+755%)	32.44	0.9611	32.44	0.7858
GPDN_2 (OURS)	6.432	102.7	31.69	0.8836	28.32	0.7747
GPDN_8 (OURS)	6.432	102.7	31.91	0.8875	28.35	0.7745

**Table 7:** Comparison with state-of-the-art methods in the B100 dataset and the Urban100 dataset. In this experiment, all are the results of dataset validation with upsampling factor ×4. Our models are trained with the cropped training dataset (DF2K_sub). GPDN_2 and GPDN_8 are our proposed models.

Methods	#FLOPs (G)	#Params (K)	B100		Urban100	
			PSNR (dB,↑)	SSIM (↑)	PSNR (dB,↑)	SSIM (↑)
Bicubic	–	–	26.19	0.6803	24.58	0.6719
RT4KSR-XXXL (Zamfir, Conde & Timofte, 2023)	3.46 (−57%)	110.4 (+7.5%)	27.11	0.7222	25.86	0.7221
CARN (Ahn, Kang & Sohn, 2018)	426 (+6,627%)	1,592 (+1,450%)	27.58	0.7349	26.07	0.7837
LapSRN (Lai et al., 2017)	370 (+5,752%)	813 (+692%)	27.32	0.7280	25.21	0.7560
SwimIR (Liang et al., 2021)	373 (+5,800%)	878 (+755%)	32.44	0.7406	32.44	0.7980
GPDN_2 (OURS)	6.432	102.7	26.98	0.7048	24.94	0.7441
GPDN_8 (OURS)	6.432	102.7	26.97	0.7042	24.84	0.7399

It is obvious that the number of learning parameters contained in the GPDN_2 and GPDN_8 is only about one-tenth of that of the Cascading Residual Network (CARN) model (*e.g*., Ahn, Kang & Sohn, 2018), and its computational requirements are only equivalent to 0.015% of CARN. This is mainly due to the lightweight nature of the GPD module. The module is mainly composed of deep convolution, maximum pooling, and point convolution. The role of deep convolution and maximum pooling is to distill pooling and extract key features, while point convolution is responsible for integrating multi-channel features. Compared with standard convolution, deep convolution can significantly reduce the number of parameters of the model. Due to the reduction in the number of parameters, the computational amount of deep convolution is correspondingly reduced. Compared with the RT4KSR-XXXL (*e.g*., Zamfir, Conde & Timofte, 2023) algorithm, although the latter is lower in computational resource consumption than GPDN_2 and GPDN_8, it is worth noting that GPDN achieves higher PSNR performance on the two benchmark datasets Set5 and Set14.

It can be inferred from Table 8 that in some specific datasets, the performance of GPDN at the perceptual level is not inferior to that of CARN and SwinIR, such as Set5 and Set14. However, the performance on B100 is not very satisfactory, mainly due to the fact that the image size of B100 after 4× downsampling is too small ( $120 \times 80$), and GPDN pursues the balance between extreme lightweight and performance. Images that are too small may not be suitable for GPDN.

**Table 8:** Comparisons with state-of-the-art methods in LPIPS. In this experiment, all are the results of dataset validation with upsampling factor ×4. The LPIPS rating indicator uses “Alex net” with a weight of “alexnet-owt-7be5be79.pth”.

Methods	#FLOPs (G)	#Params (K)	Set5	Set14	B100
			LPIPS (↓)	LPIPS (↓)	LPIPS (↓)
CARN (Ahn, Kang & Sohn, 2018)	426 (+6,627%)	1,592 (+1,450%)	0.17651	0.30283	0.38776
SwimIR (Liang et al., 2021)	373 (+5,800%)	878 (+755%)	0.16753	0.31268	0.39965
GPDN_8 (OURS)	6.432	102.7	0.17742	0.29873	0.74005

**Figure 5:**
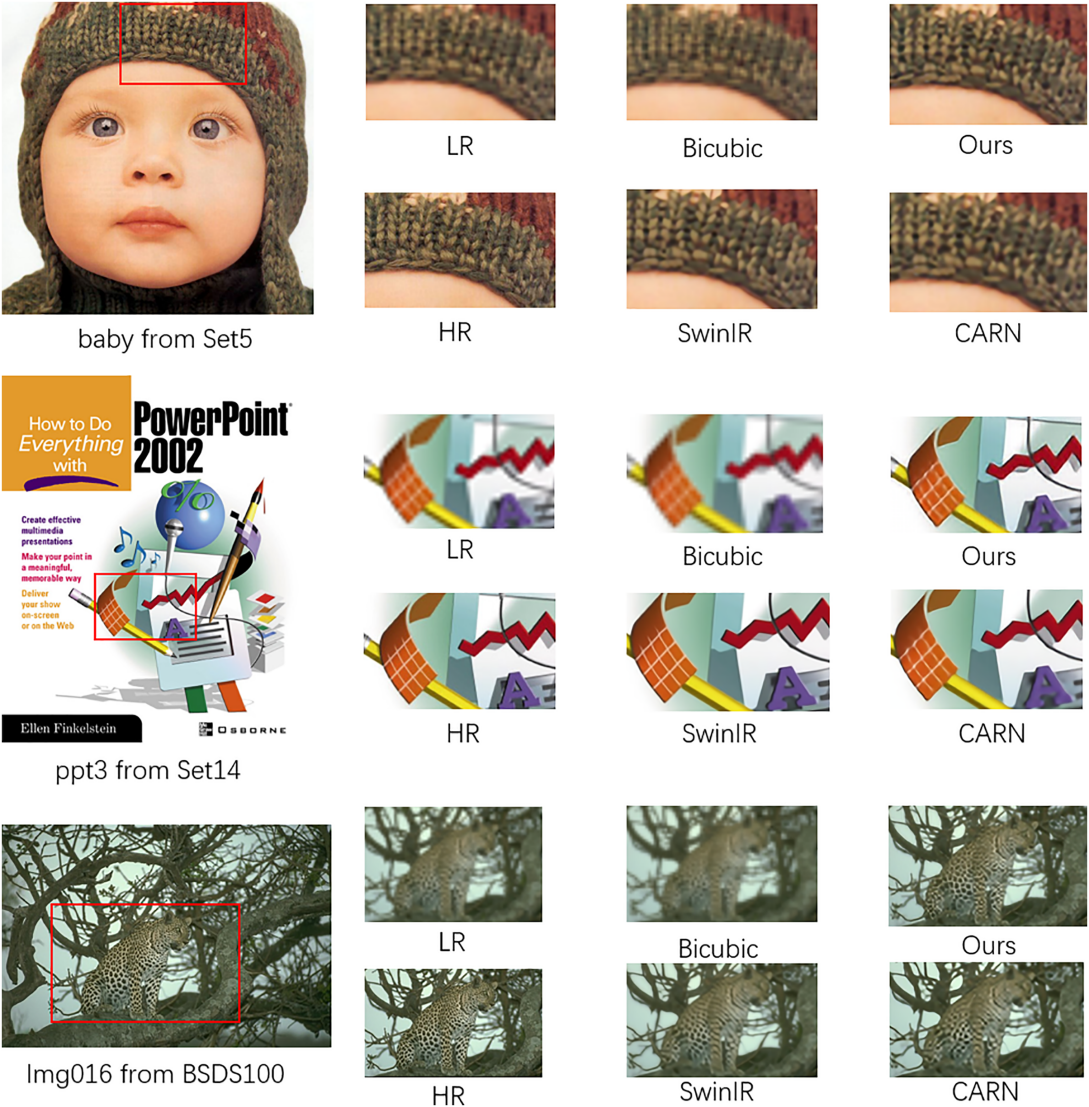
Visual comparisons for ×4 SR on the BSD100 dataset, Set5 and Set14.

Upon examining Fig. 5, it is evident that our model not only possesses the trait of being lightweight but also rivals the performance of esteemed super-resolution models such as CARN and SwinIR in certain aspects of super-resolution capabilities. In the “baby” image, it is observed that the pattern on the baby’s hat reconstructed by our model GPDN is comparable to that reconstructed by CARN, with GPDN’s reconstruction exhibiting less smearing than CARN. In the “ppt3” image, GPDN’s performance is inferior to that of CARN and SwinIR, with GPDN exhibiting a higher noise level and a less pristine output. This could be attributed to the model’s smaller parameter set, which results in a weaker capability to suppress noise. In the “Img016” image, GPDN’s reconstruction performance surpasses that of SwinIR and CARN. Upon close inspection of the cheetah’s belly pattern, the spots reconstructed by SwinIR and CARN show some merging, whereas GPDN’s reconstruction is relatively clearer with fewer merged areas.

## Conclusions

This article presents an exceptionally compact SR network, GPDN, which is characterized by its unique GPD module that efficiently extracts and integrates multi-scale feature information through a hierarchical pooling distillation strategy without introducing additional learning parameters. As a result, the visual quality and level of detail in SR reconstructed images are significantly improved. Furthermore, we analyze the specific impact of different local residual connection methods on network performance and find that selecting appropriate residual connections is crucial for optimizing the performance of SR networks. Additionally, we discover that training dataset clipping can effectively enhance the training effectiveness of SR networks. Our GPDN model not only has fewer learning parameters but also requires relatively low computing power, making it highly suitable for deployment on mobile devices to provide users with real-time high-quality image super-resolution services. In the future, we plan to further deepen our exploration and optimization of network architectures. Our focus will be on investigating how to expand the model’s adaptability to complex scenes and diverse image data while maintaining its compactness. We will study the feature differences of various types of image content when processed by the GPD module, adjust the parameters and structure of the hierarchical pooling distillation strategy accordingly, and design different feature channel modules to achieve more precise and universal multi-scale feature extraction and integration. We will also study and optimize the performance of GPDN in extreme environments. This will enhance the model’s performance in a variety of image super-resolution tasks.

## Supplemental Information

10.7717/peerj-cs.2679/supp-1 Supplemental Information 1 Testing configuration file.

10.7717/peerj-cs.2679/supp-2 Supplemental Information 2 Training startup file.

10.7717/peerj-cs.2679/supp-3 Supplemental Information 3 Testing startup file.

10.7717/peerj-cs.2679/supp-4 Supplemental Information 4 The backbone model of GPDN.

10.7717/peerj-cs.2679/supp-5 Supplemental Information 5 Environment requirements.

## Additional Information and Declarations

### Data Availability

The following information was supplied regarding data availability:

The code is available at GitHub and Zenodo:

- https://github.com/Liang123-bit/GPDN.

- Liang. (2025). GPDN’s core code. In Gradient Pooling Distillation Network for Lightweight Single Image Super-Resolution Reconstruction (1.0). PerrJ. https://doi.org/10.5281/zenodo.14649172.

The DIV2K dataset is available at: https://data.vision.ee.ethz.ch/cvl/DIV2K/.

The Flickr2K dataset is available at Zenodo: Liang. (2025). Flickr2K_hr and Flickr2K_lr_x4 [Data set]. Zenodo. https://doi.org/10.5281/zenodo.14649223.

The BSD100, Set5, Set14, Urban100 data is available at figshare: Liang, GuanJie (2024). Journal__GPDN. figshare. Journal contribution. https://doi.org/10.6084/m9.figshare.28028408.v1.

## References

[ref-1] Agustsson E, Timofte R (2017). Ntire 2017 challenge on single image super-resolution: dataset and study.

[ref-2] Ahn N, Kang B, Sohn KA (2018). Fast, accurate, and lightweight super-resolution with cascading residual network.

[ref-3] Bevilacqua M, Roumy A, Guillemot C, Alberi-Morel ML (2012). Low-complexity single-image super-resolution based on nonnegative neighbor embedding.

[ref-4] Bilecen BB, Ayazoglu M (2023). Bicubic++: slim, slimmer, slimmest-designing an industry-grade super-resolution network.

[ref-5] Chen X, Wang X, Zhou J, Qiao Y, Dong C (2023). Activating more pixels in image super-resolution transformer.

[ref-6] Dong C, Loy CC, He K, Tang X (2015). Image super-resolution using deep convolutional networks. IEEE Transactions on Pattern Analysis and Machine Intelligence.

[ref-7] Dong C, Loy CC, Tang X (2016). Accelerating the super-resolution convolutional neural network.

[ref-8] Dosovitskiy A, Beyer L, Kolesnikov A, Weissenborn D, Zhai X, Unterthiner T, Dehghani M, Minderer M, Heigold G, Gelly S, Uszkoreit S, Houlsby N (2020). An image is worth 16x16 words: transformers for image recognition at scale. https://arxiv.org/pdf/2010.11929.

[ref-9] Gunasekaran KP (2023). Ultra sharp: study of single image super resolution using residual dense network.

[ref-10] Haase D, Amthor M (2020). Rethinking depthwise separable convolutions: how intra-kernel correlations lead to improved MobileNets.

[ref-11] Haris M, Shakhnarovich G, Ukita N (2018). Deep back-projection networks for super-resolution.

[ref-12] Huang JB, Singh A, Ahuja N (2015). Single image super-resolution from transformed self-exemplars.

[ref-13] Hui Z, Gao X, Yang Y, Wang X (2019). Lightweight image super-resolution with information multi-distillation network.

[ref-14] Hui Z, Wang X, Gao X (2018). Fast and accurate single image super-resolution via information distillation network.

[ref-15] Kim J, Lee JK, Lee KM (2016a). Accurate image super-resolution using very deep convolutional networks.

[ref-16] Kim J, Lee JK, Lee KM (2016b). Deeply-recursive convolutional network for image super-resolution.

[ref-17] Kong F, Li M, Liu S, Liu D, He J, Bai Y, Chen F, Fu L (2022). Residual local feature network for efficient super-resolution.

[ref-18] Lai WS, Huang JB, Ahuja N, Yang MH (2017). Deep Laplacian pyramid networks for fast and accurate super-resolution.

[ref-19] Ledig C, Theis L, Huszár F, Caballero J, Cunningham A, Acosta A, Aitken A, Tejani A, Totz J, Wang Z, Shi W (2017). Photo-realistic single image super-resolution using a generative adversarial network.

[ref-20] Li Z, Liu Y, Chen X, Cai H, Gu J, Qiao Y, Dong C (2022). Blueprint separable residual network for efficient image super-resolution.

[ref-21] Liang J, Cao J, Sun G, Zhang K, Van Gool L, Timofte R (2021). SwinIR: image restoration using Swin transformer.

[ref-22] Lim B, Son S, Kim H, Nah S, Mu Lee K (2017). Enhanced deep residual networks for single image super-resolution.

[ref-23] Liu G, Ding Y, Li M, Sun M, Wen X, Wang B (2023). Reconstructed convolution module based look-up tables for efficient image super-resolution.

[ref-24] Liu J, Tang J, Wu G (2020). Residual feature distillation network for lightweight image super-resolution.

[ref-25] Shi W, Caballero J, Huszár F, Totz J, Aitken AP, Bishop R, Rueckert D, Wang Z (2016). Real-time single image and video super-resolution using an efficient sub-pixel convolutional neural network.

[ref-26] Simonyan K (2015). Very deep convolutional networks for large-scale image recognition. https://arxiv.org/pdf/1409.1556.

[ref-27] Sun L, Dong J, Tang J, Pan J (2023). Spatially-adaptive feature modulation for efficient image super-resolution.

[ref-28] Tai Y, Yang J, Liu X (2017). Image super-resolution via deep recursive residual network.

[ref-29] Timofte R, Agustsson E, Van Gool L, Yang MH, Zhang L (2017). Ntire 2017 challenge on single image super-resolution: methods and results. https://openaccess.thecvf.com/content_cvpr_2017_workshops/w12/papers/Timofte_NTIRE_2017_Challenge_CVPR_2017_paper.pdf.

[ref-30] Timofte R, De Smet V, Van Gool L (2015). A+: adjusted anchored neighborhood regression for fast super-resolution.

[ref-31] Zamfir E, Conde MV, Timofte R (2023). Towards real-time 4K image super-resolution.

[ref-32] Zeyde R, Elad M, Protter M (2012). On single image scale-up using sparse-representations.

[ref-33] Zhang K, Danelljan M, Li Y, Timofte R, Liu J, Tang J, Wu G, Zhu Y, He X, Xu W, Li C, Leng C, Cheng J, Wu G, Wang W, Liu X, Zhao H, Kong X, He J, Qiao Y, Dong C, Luo X, Chen L, Zhang J, Suin M, Kuldeep Purohit ANR, Li X, Lang Z, Nie J, Wei W, Zhang L, Muqeet A, Hwang J, Yang S, Kang JH, Bae S-H, Kim Y, Chen L, Zhang J, Luo X, Qu Y, Jeon G-W, Choi J-Ho, Kim J-H, Lee J-S, Marty S, Marty E, Xiong D, Chen S, Zha L, Jiang J, Gao X, Lu W, Wang H, Bhaskara V, Levinshtein A, Tsogkas S, Jepson A, Kong X, Zhao T, Zhao S, Hrishikesh PS, Puthussery D, Jiji CV, Nan N, Liu S, Cai J, Meng Z, Ding J, Ho CM, Wang X, Yan Q, Zhao Y, Chen L, Zhang J, Luo X, Chen L, Qu Y, Sun L, Wang W, Liu Z, Lan R, Umer RM, Micheloni C (2020). Aim 2020 challenge on efficient super-resolution: methods and results.

[ref-34] Zhang R, Isola P, Efros AA, Shechtman E, Wang O (2018a). The unreasonable effectiveness of deep features as a perceptual metric.

[ref-35] Zhang Y, Li K, Li K, Wang L, Zhong B, Fu Y (2018b). Image super-resolution using very deep residual channel attention networks.

[ref-36] Zhang K, Liang J, Van Gool L, Timofte R (2021). Designing a practical degradation model for deep blind image super-resolution.

[ref-37] Zhang Y, Tian Y, Kong Y, Zhong B, Fu Y (2018c). Residual dense network for image super-resolution.

[ref-38] Zhang X, Zeng H, Zhang L (2021). Edge-oriented convolution block for real-time super resolution on mobile devices.

[ref-39] Zhang X, Zhang Y, Yu F (2024). HiT-SR: hierarchical transformer for efficient image super-resolution.

[ref-40] Zhao H, Kong X, He J, Qiao Y, Dong C (2020). Efficient image super-resolution using pixel attention.

[ref-41] Zheng M, Sun L, Dong J, Pan J (2024). SMFANet: a lightweight self-modulation feature aggregation network for efficient image super-resolution.

